# (*E*)-4-[2-(2-Hy­droxy­benzo­yl)­hydra­zin­ylidene]penta­noic acid

**DOI:** 10.1107/S1600536811033988

**Published:** 2011-08-27

**Authors:** Yanling Qiao, Jichun Cui, Zhaoling Pan, Peipei Liu, Handong Yin

**Affiliations:** a Shandong Provincial Key Laboratory of Chemical Energy Storage and Novel Cell Technology, School of Chemistry and Chemical Engineering, Liaocheng University, Liaocheng 252059, People’s Republic of China; b Linyi No. 1 Middle School, Linyi 276003, People’s Republic of China; c College of Chemistry and Chemical Engineering, Liaocheng University, Shandong 252059, People’s Republic of China

## Abstract

The title mol­ecule, C_12_H_14_N_2_O_4_, adopts a *trans* configuration with respect to the C=N double bond. The amino group is involved in an intra­molecular N—H⋯O hydrogen bond. In the crystal structure, inter­molecular O—H⋯O hydrogen bonds link the mol­ecules into doubled sheets parallel to the (101) plane.

## Related literature

For the synthesis and structures of some organotin(IV) complexes of related tridentate hydrazone ligands, see: Yin *et al.* (2008 ▶).
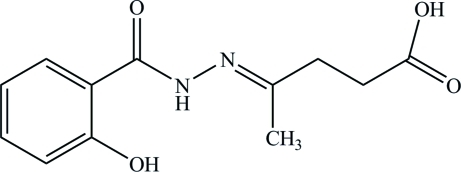


## Experimental

### 

#### Crystal data

C_12_H_14_N_2_O_4_
*M* *_r_* = 250.25
Monoclinic, 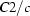

*a* = 24.445 (2) Å
*b* = 8.4683 (8) Å
*c* = 13.1204 (12) Å
β = 118.560 (1)°
*V* = 2385.6 (4) Å^3^
*Z* = 8
Mo *K*α radiation
μ = 0.11 mm^−1^
*T* = 298 K
0.45 × 0.20 × 0.17 mm

#### Data collection

Bruker SMART 1000 diffractometer
Absorption correction: multi-scan (*SADABS*; Bruker, 2001 ▶) *T*_min_ = 0.954, *T*_max_ = 0.982
5738 measured reflections
2092 independent reflections
1013 reflections with *I* > 2σ(*I*)
*R*_int_ = 0.062

#### Refinement

*R*[*F* ^2^ > 2σ(*F* ^2^)] = 0.048
*wR*(*F* ^2^) = 0.114
*S* = 1.00
2092 reflections
164 parameters
H-atom parameters constrained
Δρ_max_ = 0.23 e Å^−3^
Δρ_min_ = −0.26 e Å^−3^

### 

Data collection: *SMART* (Bruker, 2007 ▶); cell refinement: *SAINT* (Bruker, 2007 ▶); data reduction: *SAINT*; program(s) used to solve structure: *SHELXS97* (Sheldrick, 2008 ▶); program(s) used to refine structure: *SHELXL97* (Sheldrick, 2008 ▶); molecular graphics: *SHELXTL* (Sheldrick, 2008 ▶); software used to prepare material for publication: *SHELXTL*.

## Supplementary Material

Crystal structure: contains datablock(s) I, global. DOI: 10.1107/S1600536811033988/cv5127sup1.cif

Structure factors: contains datablock(s) I. DOI: 10.1107/S1600536811033988/cv5127Isup2.hkl

Supplementary material file. DOI: 10.1107/S1600536811033988/cv5127Isup3.cml

Additional supplementary materials:  crystallographic information; 3D view; checkCIF report

## Figures and Tables

**Table 1:** Hydrogen-bond geometry (Å, °)

*D*—H⋯*A*	*D*—H	H⋯*A*	*D*⋯*A*	*D*—H⋯*A*
O4—H4⋯O2^i^	0.82	1.91	2.679 (3)	155
O1—H1⋯O3^ii^	0.82	1.80	2.570 (3)	155
N2—H2⋯O4	0.86	1.95	2.635 (3)	136

Symmetry codes: (i) 
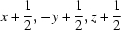
; (ii) 
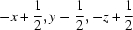
.

**Fig. 1.**
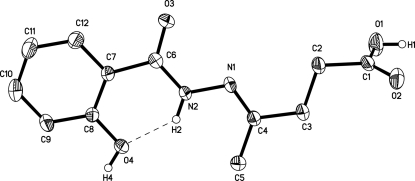
The molecular structure of (I), showing 50% probability displacement ellipsoids. Dashed line denotes hydrogen bond.

**Fig. 2.**
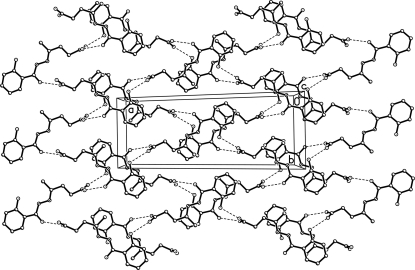
A portion of the crystal packing, showing hydrogen-bonded (dashed lines) two-dimensional network. H atoms have been omitted for clarity.

## supplementary crystallographic information

### Comment

Recently, we have reported some organotin(IV) complexes with hydrazone ligands (Yin *et al.*,  2008). As an extension of our work on the structural characterization of hydrazone compounds, the title compound, (I), is reported here.

In the title compound, (I), the N1═C4 bond length of 1.276 (3) Å is a typical double bond value, while the N2—C6 [1.337 (3) Å] and N1—N2 [1.390 (3) Å] bonds are intermediate between double and single bonds because of conjugation effects in the molecule.

In the crystal structure, intermolecular O—H···O hydrogen bonds (Table 1) link the molecules into doubled sheets parallel to (101) plane.

### Experimental

Compound (I) was synthesized by the reaction of 2-hydroxybenzohydrazide (10 mmol) with 4-oxopentanoic acid (10 mmol). Single crystals of (I) suitable for X-ray diffraction were obtained by slow evaporation of a methanol solution.

### Refinement

The H atoms were positioned geometrically, with methyl C—H distances of 0.96 Å, methylene C—H distances of 0.93 Å, aromatic  C—H distances of 0.93 Å, N—H distances of 0.86 Å and O—H distances of 0.82 Å, and refined as riding on their parent atoms, with *U*_iso_(H) = 1.2-1.5 *U*_eq_ of the parent atom.

### Crystal data

**Table:**

C_12_H_14_N_2_O_4_	*F*(000) = 1056
*M**_r_* = 250.25	*D*_x_ = 1.394 Mg m^−^^3^
Monoclinic, *C*2/*c*	Mo *K*α radiation, λ = 0.71073 Å
*a* = 24.445 (2) Å	Cell parameters from 770 reflections
*b* = 8.4683 (8) Å	θ = 2.6–20.7°
*c* = 13.1204 (12) Å	µ = 0.11 mm^−^^1^
β = 118.560 (1)°	*T* = 298 K
*V* = 2385.6 (4) Å^3^	Block, colourless
*Z* = 8	0.45 × 0.20 × 0.17 mm

### Data collection

**Table:**

Bruker SMART 1000 diffractometer	2092 independent reflections
Radiation source: fine-focus sealed tube	1013 reflections with *I* > 2σ(*I*)
graphite	*R*_int_ = 0.062
φ and ω scans	θ_max_ = 25.0°, θ_min_ = 1.9°
Absorption correction: multi-scan (*SADABS*; Bruker, 2001)	*h* = −28→14
*T*_min_ = 0.954, *T*_max_ = 0.982	*k* = −10→9
5738 measured reflections	*l* = −15→15

### Refinement

**Table:**

Refinement on *F*^2^	Primary atom site location: structure-invariant direct methods
Least-squares matrix: full	Secondary atom site location: difference Fourier map
*R*[*F*^2^ > 2σ(*F*^2^)] = 0.048	Hydrogen site location: inferred from neighbouring sites
*wR*(*F*^2^) = 0.114	H-atom parameters constrained
*S* = 1.00	*w* = 1/[σ^2^(*F*_o_^2^) + (0.0385*P*)^2^] where *P* = (*F*_o_^2^ + 2*F*_c_^2^)/3
2092 reflections	(Δ/σ)_max_ < 0.001
164 parameters	Δρ_max_ = 0.23 e Å^−^^3^
0 restraints	Δρ_min_ = −0.26 e Å^−^^3^

### Fractional atomic coordinates and isotropic or equivalent isotropic displacement parameters (Å^2^)

**Table:**

	*x*	*y*	*z*	*U*_iso_*/*U*_eq_	
N1	0.39247 (9)	0.4503 (3)	0.29554 (19)	0.0352 (6)	
N2	0.45430 (9)	0.4964 (3)	0.34082 (18)	0.0351 (6)	
H2	0.4823	0.4263	0.3536	0.042*	
O1	0.18399 (10)	0.3011 (3)	0.2303 (2)	0.0700 (7)	
H1	0.1496	0.2591	0.2013	0.105*	
O2	0.17818 (9)	0.2415 (2)	0.0602 (2)	0.0586 (7)	
O3	0.43347 (9)	0.7535 (2)	0.35295 (18)	0.0528 (6)	
O4	0.57296 (8)	0.4200 (2)	0.43211 (16)	0.0447 (6)	
H4	0.6050	0.3692	0.4522	0.067*	
C1	0.20548 (13)	0.2976 (3)	0.1566 (3)	0.0429 (8)	
C2	0.26898 (12)	0.3703 (3)	0.2044 (3)	0.0485 (9)	
H2A	0.2796	0.4225	0.2774	0.058*	
H2B	0.2689	0.4491	0.1506	0.058*	
C3	0.31687 (12)	0.2454 (3)	0.2235 (3)	0.0445 (8)	
H3A	0.3143	0.1641	0.2732	0.053*	
H3B	0.3062	0.1969	0.1494	0.053*	
C4	0.38293 (12)	0.3024 (3)	0.2768 (2)	0.0367 (8)	
C5	0.43127 (12)	0.1791 (3)	0.3004 (3)	0.0488 (9)	
H5A	0.4466	0.1895	0.2456	0.073*	
H5B	0.4133	0.0763	0.2931	0.073*	
H5C	0.4651	0.1924	0.3776	0.073*	
C6	0.47095 (13)	0.6478 (4)	0.3649 (2)	0.0352 (7)	
C7	0.53770 (12)	0.6864 (3)	0.4048 (2)	0.0333 (7)	
C8	0.58579 (12)	0.5776 (3)	0.4357 (2)	0.0337 (7)	
C9	0.64610 (13)	0.6295 (4)	0.4698 (2)	0.0461 (8)	
H9	0.6779	0.5562	0.4897	0.055*	
C10	0.65900 (15)	0.7874 (4)	0.4743 (3)	0.0570 (10)	
H10	0.6994	0.8208	0.4969	0.068*	
C11	0.61268 (15)	0.8956 (4)	0.4456 (3)	0.0583 (10)	
H11	0.6215	1.0029	0.4492	0.070*	
C12	0.55293 (13)	0.8455 (3)	0.4115 (2)	0.0457 (8)	
H12	0.5217	0.9205	0.3922	0.055*	

### Atomic displacement parameters (Å^2^)

**Table:**

	*U*^11^	*U*^22^	*U*^33^	*U*^12^	*U*^13^	*U*^23^
N1	0.0219 (14)	0.0424 (16)	0.0391 (16)	−0.0016 (11)	0.0127 (11)	0.0005 (12)
N2	0.0233 (13)	0.0326 (14)	0.0479 (16)	−0.0001 (11)	0.0160 (12)	−0.0001 (12)
O1	0.0452 (14)	0.0932 (19)	0.0764 (18)	−0.0259 (12)	0.0329 (13)	−0.0195 (15)
O2	0.0395 (13)	0.0633 (16)	0.0669 (17)	−0.0156 (11)	0.0206 (12)	−0.0133 (13)
O3	0.0373 (12)	0.0370 (13)	0.0851 (17)	0.0070 (10)	0.0301 (12)	0.0027 (12)
O4	0.0293 (11)	0.0377 (13)	0.0612 (15)	0.0056 (9)	0.0167 (10)	0.0010 (11)
C1	0.0274 (18)	0.0371 (19)	0.061 (3)	0.0012 (14)	0.0184 (18)	0.0041 (18)
C2	0.0297 (17)	0.0404 (18)	0.070 (2)	−0.0044 (14)	0.0190 (16)	0.0016 (17)
C3	0.0279 (17)	0.0460 (19)	0.057 (2)	−0.0046 (14)	0.0180 (16)	−0.0035 (16)
C4	0.0302 (17)	0.0376 (19)	0.042 (2)	−0.0009 (14)	0.0172 (15)	0.0004 (15)
C5	0.0409 (19)	0.0369 (18)	0.070 (2)	−0.0001 (14)	0.0278 (17)	−0.0053 (16)
C6	0.0304 (17)	0.040 (2)	0.0349 (19)	0.0011 (15)	0.0157 (14)	0.0011 (15)
C7	0.0273 (16)	0.0350 (18)	0.0343 (18)	−0.0020 (13)	0.0122 (14)	−0.0014 (14)
C8	0.0319 (17)	0.0339 (18)	0.0371 (19)	−0.0036 (14)	0.0179 (15)	−0.0028 (14)
C9	0.0299 (18)	0.054 (2)	0.054 (2)	−0.0030 (15)	0.0196 (17)	−0.0033 (17)
C10	0.038 (2)	0.069 (3)	0.058 (2)	−0.0189 (19)	0.0188 (18)	−0.005 (2)
C11	0.053 (2)	0.047 (2)	0.067 (3)	−0.0174 (18)	0.022 (2)	−0.0002 (18)
C12	0.042 (2)	0.039 (2)	0.049 (2)	−0.0028 (15)	0.0163 (17)	0.0008 (16)

### Geometric parameters (Å, °)

**Table:**

N1—C4	1.276 (3)	C3—H3B	0.9700
N1—N2	1.390 (3)	C4—C5	1.494 (3)
N2—C6	1.337 (3)	C5—H5A	0.9600
N2—H2	0.8600	C5—H5B	0.9600
O1—C1	1.303 (3)	C5—H5C	0.9600
O1—H1	0.8200	C6—C7	1.492 (3)
O2—C1	1.209 (3)	C7—C12	1.390 (3)
O3—C6	1.236 (3)	C7—C8	1.393 (3)
O4—C8	1.366 (3)	C8—C9	1.392 (3)
O4—H4	0.8200	C9—C10	1.368 (4)
C1—C2	1.501 (4)	C9—H9	0.9300
C2—C3	1.507 (3)	C10—C11	1.363 (4)
C2—H2A	0.9700	C10—H10	0.9300
C2—H2B	0.9700	C11—C12	1.374 (4)
C3—C4	1.500 (3)	C11—H11	0.9300
C3—H3A	0.9700	C12—H12	0.9300
			
C4—N1—N2	114.8 (2)	H5A—C5—H5B	109.5
C6—N2—N1	121.1 (2)	C4—C5—H5C	109.5
C6—N2—H2	119.5	H5A—C5—H5C	109.5
N1—N2—H2	119.5	H5B—C5—H5C	109.5
C1—O1—H1	109.5	O3—C6—N2	122.8 (3)
C8—O4—H4	109.5	O3—C6—C7	120.4 (3)
O2—C1—O1	124.6 (3)	N2—C6—C7	116.8 (2)
O2—C1—C2	122.8 (3)	C12—C7—C8	117.4 (3)
O1—C1—C2	112.5 (3)	C12—C7—C6	116.7 (2)
C1—C2—C3	110.3 (2)	C8—C7—C6	125.9 (3)
C1—C2—H2A	109.6	O4—C8—C9	120.7 (2)
C3—C2—H2A	109.6	O4—C8—C7	119.2 (2)
C1—C2—H2B	109.6	C9—C8—C7	120.1 (3)
C3—C2—H2B	109.6	C10—C9—C8	120.6 (3)
H2A—C2—H2B	108.1	C10—C9—H9	119.7
C4—C3—C2	115.4 (2)	C8—C9—H9	119.7
C4—C3—H3A	108.4	C11—C10—C9	120.1 (3)
C2—C3—H3A	108.4	C11—C10—H10	120.0
C4—C3—H3B	108.4	C9—C10—H10	120.0
C2—C3—H3B	108.4	C10—C11—C12	119.7 (3)
H3A—C3—H3B	107.5	C10—C11—H11	120.1
N1—C4—C5	126.3 (2)	C12—C11—H11	120.1
N1—C4—C3	117.5 (2)	C11—C12—C7	122.1 (3)
C5—C4—C3	116.2 (2)	C11—C12—H12	119.0
C4—C5—H5A	109.5	C7—C12—H12	119.0
C4—C5—H5B	109.5		

### Hydrogen-bond geometry (Å, °)

**Table:**

*D*—H···*A*	*D*—H	H···*A*	*D*···*A*	*D*—H···*A*
O4—H4···O2^i^	0.82	1.91	2.679 (3)	155.
O1—H1···O3^ii^	0.82	1.80	2.570 (3)	155.
N2—H2···O4	0.86	1.95	2.635 (3)	136.

Symmetry codes: (i) *x*+1/2, −*y*+1/2, *z*+1/2; (ii) −*x*+1/2, *y*−1/2, −*z*+1/2.
